# Testing the Accuracy of Pont’s Index in Diagnosing Maxillary Transverse Discrepancy as Compared to the University of Pennsylvania CBCT Analysis

**DOI:** 10.3390/dj10020023

**Published:** 2022-02-04

**Authors:** Dana Feştilă, Aurelia Magdalena Enache, Evelyn Beatrix Nagy, Mihaela Hedeşiu, Mircea Ghergie

**Affiliations:** 1 Department of Orthodontics and Dentofacial Orthopedics, Faculty of Dental Medicine, University of Medicine and Pharmacy “Iuliu Haţieganu”, 400349 Cluj-Napoca, Romania; dana.festila@gmail.com (D.F.); mhedesiu@umfcluj.ro (M.H.); mirceaghergie@yahoo.com (M.G.); 2 Department of Orthodontics and Dentofacial Orthopedics, Faculty of Dental Medicine, “C. Davilla” University of Medicine and Pharmacy, 020021 Bucharest, Romania; 3 B-Line Dent, Str. Observatorului nr. 7 ap. 35, 400500 Cluj-Napoca, Romania

**Keywords:** orthodontics, maxilla, transverse discrepancy, CBCT, diagnosis

## Abstract

**Introduction:** Assessment of maxillary transverse discrepancy requires an accurate tool in order to implement the appropriate treatment plan. **Aim:** To evaluate the accuracy of Pont’s Index in confirming a transverse maxillary deficiency by comparing its results with the corresponding results yielded by the University of Pennsylvania CBCT analysis. **Material and methods:** The study was performed on dental casts and CBCT’s of 60 randomly selected patients by adopting a cluster sampling method. The results of Pont’s Index and University of Pennsylvania CBCT analysis were analyzed through McNemar’s test using Excel Data Analysis, and the accuracy indicators for Pont’s Index were computed using MedCalc Ltd. **Results:** Mc Nemar’s test revealed a *p*-value of 0.85. The accuracy indicators of Pont’s Index were: sensitivity: 69%; specificity: 16.6%; positive predictive value: 65%; negative predictive value: 18.75%; positive likelihood ratio: 0.83; negative likelihood ratio: 1.86; and accuracy: 53.28%. **Conclusion:** Due to the fact that CBCT is not used on a daily basis and Pont’s Index has a relatively high sensitivity (69%) making it suitable to detect patients with a narrow maxilla, assessment of the maxillary deficiency on CBCT can be recommended for cases were the midpalatal suture maturation should be evaluated.

## 1. Introduction

It is widely known that maxillary transverse discrepancy (MTD) is a common trait of malocclusions; hence its accurate evaluation for appropriate treatment is mandatory to reduce the side effects of different modalities of maxillary expansion and to obtain good dental and skeletal stability and a balanced smile [1,2,3]. Besides clinical evaluation, the diagnosis of MTD can be made lighter and more accurate by using dental casts, X-rays examinations, and 3D imaging [1,2,3,4]. As the diagnosis of MTD is often challenging, one should use at least two of the previously mentioned methods. As it is known, the roll and yaw of the jaws and dentition affect the dentofacial transverse relationship; hence it is of great importance to distinguish between skeletal and dental maxillary deficiency [5]. Besides dental compensation of a skeletal MTD by the buccolingual inclination of the posterior teeth, the anterioposterior component of malocclusion can classify the MTD in real or relative because a class II relationship can mask an MTD, whereas a class III can accentuate it [6]. After having performed clinical examination of the occlusion in the transverse plane and of the palatal vault shape and appearance, since the most frequent clinical signs of MTD are uni-or bilateral posterior crossbite, crowding, V-shape, and narrow palate, dental casts examination is the first step to confirm the MTD by measuring Pont’s and McNamara Index [7,8]. However, Pont’s Index has some limitations determined by abnormal variations of the mesiodistal width of the incisors, mesiodistal position, and buccolingual inclination of the premolars and molars (as a compensation of the maxillary skeletal deficiency).

2D X-ray examination (posteroanterior cephalogram) was considered a more accurate method to assess MTD, but it also has limitations regarding the accuracy of landmark placement and expertise of the practitioner to determine the landmarks [9].

New 3D imaging is a feasible diagnostic tool in patient assessment, as digital models and CT scans have considerably improved the diagnostic method for assessing dental and skeletal maxillary deficiency [10]. Novel technology and the article by Tamburrino et al. (2010) opened a new direction of research towards the contribution of cone-beam computed tomography (CBCT) in the evaluation and diagnosis of the yet scarcely researched transverse dimension of the maxilla [1,4]. To date, the measurement of the transverse discrepancy of the jaws is rarely among the case types in which CBCT could be justified for optimal treatment outcomes [4]. Exploring this area could bring new enlightenments about this perspective.

Although CBCT entered dentistry in 1998, scientific evidence of improving treatment planning and treatment outcomes in orthodontics has emerged only recently [4]. 3D technology is undergoing rapid evolution, yet several aspects, such as indications for CBCT imaging and protocols regarding diagnosis, need further research [2,4]. Nevertheless, the novel technology already allowed orthodontists to obtain measurements without distortions, thus enabling research on the diagnosis of maxillary narrowing using CBCT imaging by calculating the difference in width between the maxilla (Mx-Mx) and the mandible (WALA-WALA) [1]. Two gold standards were established, these being the University of Pennsylvania CBCT analysis (UPCBCT analysis) and center of alveolar crest (CAC) technique [11,12].

One of the benefits of CBCT lies in its volumetric information that overcomes the limitations of 2D imaging that has distortion, magnification, incorrect head position, and superimposition [4]. The advantage of the cone-beam computer tomography proposed by the UPCBCT analysis emerges from the ease of landmark identification, which no longer enforces the landmark selection for measuring the widths of the jaws [1]. Another considerable benefit of CBCT to diagnose MTD is the use of differential data to analyze the lower arch expansion. Since RME determines a slight buccal inclination and a slight increase in the intra-arch width between mandibular posterior teeth, with no difference between tooth-borne or bone-borne appliance, one has to consider it for the treatment plan [13,14].

Pont’s Index (PI) assesses MTD by comparing the ideal inter-premolar (IP) and inter-molar (IM) distances to the calculated IP and IM. The advantages of PI are the lack of radiation, ease of use, and low cost. Although Pont’s Index (PI) is widely used, further studies on various ethnic groups are needed to take into consideration their craniofacial parameters [15].

The purpose of the current study was to evaluate the accuracy of PI as a diagnostic method in confirming MTD by comparing the obtained results with the corresponding results yielded by the UPCBCT analysis, thus assessing the importance and the usefulness of the two tools in the diagnostic process.

The main objective was to test a link between the test results obtained by PI and the presence or absence of MTD as indicated by the gold standard, the UPCBCT analysis.

The second objective was to quantify the importance of the correlation by computing accuracy indicators.

The following hypotheses were considered:

$H_{0}$: The difference between the results of PI and UPCBCT analysis in the accuracy of diagnosing MTD is not statistically significant.

$H_{1}$: The difference between the results of PI and UPCBCT analysis in the accuracy of diagnosing MTD is statistically significant, with PI being less accurate in diagnosing a transverse maxillary deficiency than the UPCBCT analysis.

The results of the present study were meant to confirm one of the hypotheses and deny the other.

## 2. Materials and Methods

A retrospective study was conducted on a sample of dental casts and CBCT scans of 60 patients with MTD from two orthodontic offices (two cities Cluj Napoca and Satu Mare) randomly selected by adopting a convenience and a cluster sampling method and in accordance with the following inclusion criteria: 1. no orthodontic treatment before; 2. acceptable quality CBCT images with clearly identifiable structures of the maxilla and mandible (see Appendix A for further details regarding CBCT characteristics); 3. good-quality dental casts without any deterioration that could hinder the measurements; 4. dental cast and CBCT image obtained in the same week.

The exclusion criteria were the absence of the reference teeth: upper incisors, first upper premolar and first permanent molars, macro- or microdontia, and presence of deciduous teeth.

A total of 158 subjects were excluded from an initial number of 218 cases considered for the present study. Frequent criteria for exclusions were the dental compensation, absence of reference teeth (upper lateral incisors, 1st maxillary and mandibular molars, and maxillary premolars), deficient records (absent casts or CBCT scans), and treatment already started at the time of performing the CBCT scans.

For each patient, the transverse dimension of the maxilla was assessed by a single trained investigator supervised by the authors responsible for this role, both on dental cast using PI with a digital caliper with sharp beaks and on CBCT images by the UPCBCT analysis with Planmeca Romexis Viewer 6.0.1.812 software. The results were calculated after measurements were taken for both tests, and the thresholds for classifying the results were pre-specified.

**Figure 1:**
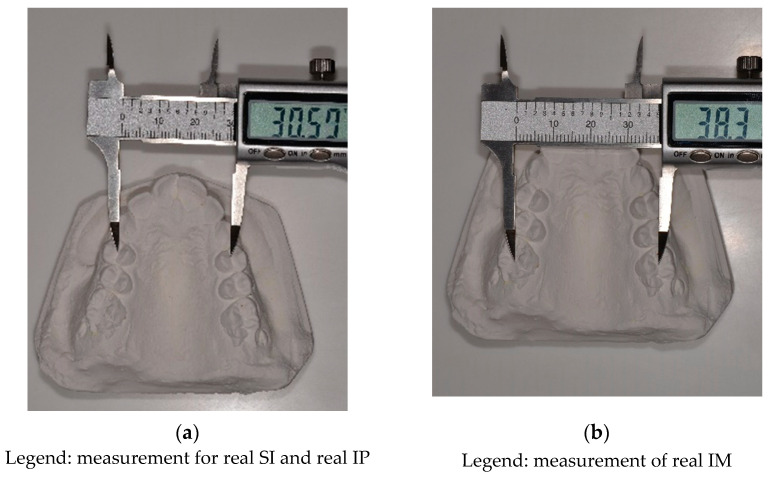
Methods of measuring: (**a**) SI and IP with the digital caliper by placing the beaks in the center of the occlusal groove of the first maxillary premolars; (**b**) IM in the center of the occlusal surface of the first permanent molars.

To calculate the ideal value of PI for a particular patient, the sum of the mesiodistal diameter of the four upper incisors (SI) was first measured (Figure 1a). Secondly, the ideal inter-premolar distance (IP) and the ideal inter-molar distance (IM) were calculated using the following formulae [16]:

Ideal IP = SI × 100/80 (premolar arch width)

Ideal IM = SI × 100/64 (molar arch width)

The real premolar and molar arch width were measured with the digital caliper by placing the beaks in the center of the occlusal groove of the first maxillary premolars and the center of the occlusal surface of the first permanent molars (Figure 1b). The measured value is subtracted from the calculated value to assess the presence of a maxillary deficiency. If the measured values are smaller than the calculated ones, then a narrowing of the maxilla is indicated by PI at the level of the premolars or molars. A 2 mm difference between the calculated and measured values is allowed. A mild maxillary narrowing is diagnosed when the difference is between 2 and 4 mm. When the difference is between 4 and 6 mm, a medium narrowing is present. A severe narrowing occurs when the difference is between 6 and 10 mm, while a very severe narrowing is present when the difference is more than 10 mm. As mentioned before in the introduction, Pont’s Index has some drawbacks that may induce errors in results interpretation. The buccal inclination of the posterior teeth determines a higher value for real IP and IM that masks a true skeletal MTD. Also, the abnormal variations of mesiodistal size of the incisors, as hypoplastic lateral incisor or Bolton discrepancy, can modify the value of SI and the ideal value of IP and IM, which leads to a false interpretation of the results.

**Figure 2:**
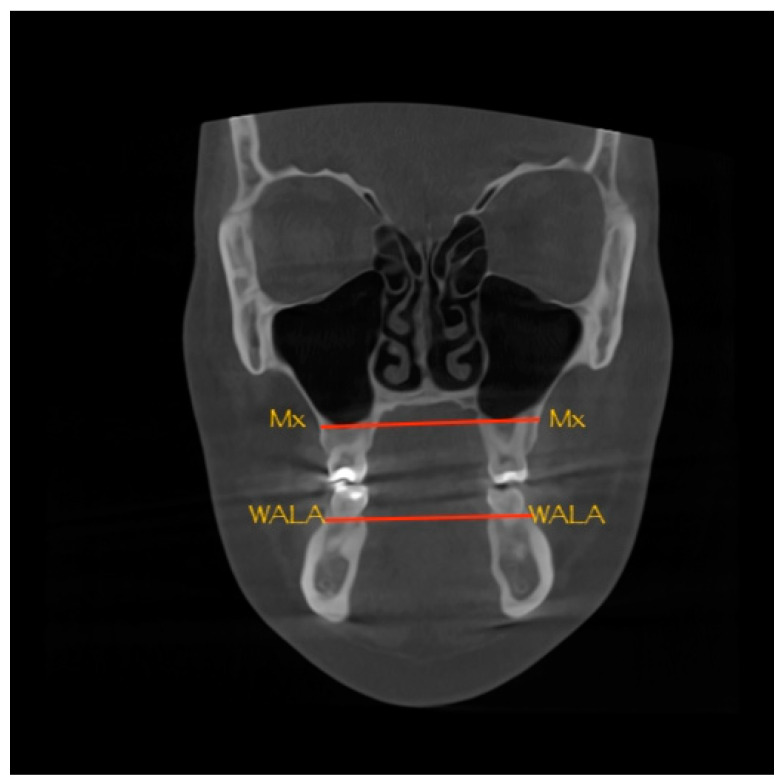
UPCBCT analysis: Mx-Mx and WALA-WALA distances.

For calculating the ideal value of UPCBCT analysis, the Mx-Mx (Figure 2) and the WALA-WALA (Figure 2) distances were measured on the CBCT scans. To calculate the difference in width between the maxilla and the mandible, the mandibular width was subtracted from the maxillary width then the measured difference was subtracted from 5 to determine the amount of expansion needed, expressed in millimeters [1,17].

### Statistical Analysis

The results were analyzed using McNemar’s test computed using appropriate functions on Excel Data Analysis version 16.16.27 (201012) and the accuracy indicators calculated using the online version of © 2022 MedCalc Software Ltd. [18]. As both tests were performed on the same series of subjects (matched groups), thus obtaining paired data, McNemar’s test was the best choice to correlate the test results of PI and the presence or absence of MTD as indicated by the results of UPCBCT analysis. In order to reject the null hypothesis ($H_{0}),\;$differences were considered statistically significant if the *p*-value was less than 0.05 [19].

To quantify the link between the test results of PI and the presence or absence of MTD, several diagnostic accuracy indicators needed to be computed. A contingency table based on a pivot table was used to calculate: sensitivity (Se), specificity (Sp), accuracy, positive likelihood ratio (+LR), negative likelihood ratio (−LR), positive predictive value (PPV), and negative predictive value (NPV) [20,21,22].

## 3. Results

A total number of 218 patients were considered for the study, from which 158 were eliminated, leaving a final total of 60 patients for the present study. The exclusion rate was 72.5%. Of the target sample, 30.27% were eliminated due to dental compensation and missing reference teeth (upper lateral incisors, 1st maxillary and mandibular molars, and maxillary premolars), 20.64% were eliminated due to incomplete records, and a further 9.17% were eliminated due to having started the orthodontic treatment before the initial CBCT was taken or having macro-, microdontia. The preliminary power was 0.8.

Table 1 depicts the results of the measurements of the transverse dimension of the maxilla in the current study’s participants. The column “Results match” indicates whether the results of the two diagnostic methods match. An “N” indicates results that do not match. A “Y” indicates that both UPCBCT analysis and PI indicate a narrow maxilla. A matching result occurred in three cases:

⚬ When UPCBCT analysis indicates MTD and PI indicates MTD in the premolar region (IP);
⚬ When UPCBCT analysis indicates MTD and PI indicates MTD in the molar region (IM);
⚬ When UPCBCT analysis indicates MTD and PI indicates MTD both in the premolar (IP) and molar (IM) regions.

In contrast to UPCBCT analysis, PI can have four test result types (TP, FP, TN, FN) as depicted in the last column of Table 1, which are the basis for computing the accuracy indicators. A summary of the four diagnostic types was included in the contingency table (Table 2). Table 3 summarizes the resulted values of the accuracy indicators.

In order to refute or accept the null hypothesis, McNemar’s test was computed using functions in an excel sheet (Table 4).

## 4. Discussions

The disadvantages of UPCBCT analysis resulting from the CBCT technology lead to the need for a preliminary risk–benefit analysis (especially in children) and for an assessment of the accuracy of alternative diagnostic methods (PI) [4].

Comparisons were made between the results of the two diagnostic methods for MTD, PI, and UPCBCT analysis. The statistical test used to assess the difference between the two diagnostic methods was the McNemar’s test, together with accuracy indicators. McNemar’s test revealed a *p*-value of 0.85, indicating that the differences between the results of the two methods are not statistically significant, which could be explained by an insufficient number of tested subjects. Therefore, the null hypothesis $H_{0}$ of the current study has to be accepted.

Furthermore, a sensitivity of 69% indicates a probability of 0.69 for a patient with a maxillary transverse deficiency to test positively using PI. A specificity of 16.6% may indicate a probability of 0.166 for a patient without a maxillary deficiency to test negatively using PI. These are only estimates for sensitivity and specificity as they are based on a subset of subjects from the target population; if a different subset of subjects was tested or the same subjects was tested at different times, the estimates of sensitivity and specificity would be numerically different [20,22,23].

Cases of MTD in children or when the midpalatal suture is still active can be treated with numerous appliances [24]; a diagnostic test with high sensitivity is required to treat subjects with narrow maxilla. The UPCBCT analysis would be suitable as it represents the current gold standard [11,12]; however, due to its risk of radiation, another diagnostic test with higher sensitivity should be considered. The present study found that in comparison with the UPCBCT analysis, PI has a sensitivity of 69%, making it suitable for diagnosing a transverse maxillary deficiency in minors [20].

On the other hand, the treatment of MTD in adults, where the midpalatal suture is closed, might require micro-implants-assisted rapid palatal expansion or surgically assisted rapid maxillary expansion, suggesting the need for a diagnostic test with high specificity to avoid treating patients who do not have a narrow maxilla [20,25]. In these situations, diagnosis with the UPCBCT analysis is justified despite the risk of ionizing radiation [20].

A PPV of 65.9% indicates a probability of 0.659 for subjects who tested positive with PI to have MTD, whereas an NPV of 18.75% indicates a probability of 0.1875 for subjects who tested negative with PI to not have MTD. The increased percentage of the PPV and the relatively low percentage of the NPV can be explained by the high estimated prevalence of MTD, equaling 70% among the target population in the present study. The higher the prevalence of the disease (MTD), the more the certainty of a positive test result indicating the presence of the disease increases, thus having a high PPV. Inversely, the lower the prevalence, the more the certainty of a negative test result indicating the absence of MTD increases, meaning a high NPV. Therefore, PPV and NPV estimates within the present study cannot be applied to other populations without knowing the prevalence of MTD in those populations [20,22].

The LR indicates to which extent a positive or negative test result can change the likelihood that a subject has the disease. The +LR of PI is +0.83, indicating a 1-fold increase in the odds of having MTD in a patient with a positive result. The higher the +LR, the more informative PI is. On the other hand, a +LR of 1.0 means that PI is useless as the odds of having MTD has not changed after the PI (a 1-fold increase in the odds means the odds have not changed). Nevertheless, the −LR for PI is +1.86, indicating a 2-fold decrease in the odds of having MTD in a patient with a negative result. The smaller the −LR, the more informative the test. The use of a diagnostic test is based on the importance of ruling in or ruling out the disease, since an +LR greater than 10 or an −LR lower than 0.1 provide convincing diagnostic evidence. However, single studies are usually less informative regarding LRs than high-quality systematic reviews, indicating the inclusion of the present study in systematic reviews together with other similar studies and the need for further research [26].

The accuracy of the PI as compared to the UPCBCT analysis in the current study was 53.28%, meaning that the PI has 53.28% correct results among all the test results [27].

The key inclusion and exclusion criteria, along with the materials and methods, were clearly described, leading to the replicability of the present study, thus increasing its external validity. The diagnostic tests can be reproduced by trained personnel, and it further depends on the availability of CBCT technology in the area. Although in the present study, comparisons were made between the CBCT scans of one patient and the dental cast of the same patient, CBCT characteristics were provided in Appendix A. The relevance of the CBCT characteristics within the context of the present study’s design could be the subject of further research.

Over- or under- interpretation of the reference standard was avoided by calculating the results only after all measurements were taken for both diagnostic tests and after pre-specified threshold values were used to classify the results. The present study was based on the guiding principles of the STARD 2015 statement (STAndards for Reporting Diagnostic accuracy studies) [28].

A cluster sampling method was used to select subjects for the present study, the sample being representative of the population, as there were equal chances for people to choose one or the other of the two orthodontic office (Cluj-Napoca and Satu Mare, two Romanian localities). By adopting a cluster sampling method, a random inclusion of various degrees of MTD was achieved, leading to the generalizability of the findings to the target population [28].

Bias could arise from the fact that there was a single trained examiner, leading to low inter-examiner reliability. Moreover, as the measurements were not performed two times with a specific time difference, the intra-examiner reliability could be one of the study’s limitations [28].

The present study is innovative as no other study compares the UPCBCT analysis to PI. Even though the current study did not find a statistically significant difference between PI and UPCBCT analysis (*p* > 0.05), the literature review by Kathiravan concluded that PI cannot be used to predict maxillary arch width reliably [15]. A further disadvantage of PI is that the measurements can be negatively affected by dental compensation (buccolingual inclination) leading to underdiagnosis of skeletal maxillary constriction. Nevertheless, Agnihotri and Gulati support the use of PI as they found in their recent study a definite correlation between the width of the arch and the width of the four maxillary incisors within the Northern Indian population [29].

## 5. Conclusions

Within the limitations of the present study and based on its results, the following conclusions can be summarized:

1. There was no statistically significant difference between the two diagnostic methods as the null hypothesis was accepted.
2. Since CBCT is not a daily used investigation and PI has a relatively high sensitivity (69%), PI is suitable to detect MTD. However, assessment of MTD on CBCT can be recommended for cases where the midpalatal suture maturation should also be evaluated.

## Figures and Tables

**Table 1:** Comparative evaluation of a transverse maxillary deficiency using the two methods: PI and UPCBCT analysis.

Case Number	Pont’s Index		UPCBCT Results (mm)	Results Match * YES(Y)/NO(N)	TP/FP/TN/FN **
	Calculated–Measured IP (mm)	Calculated–Measured IM (mm)			
1	−1.9	1.75	−3.4	Y	TN
2	2.9875	2.599375	0.19	Y	TP
3	2.2175	1.296875	−2.2	N	FP
4	1.58	6.93	1	Y	TP
5	5.8875	4.391875	4	Y	TP
6	3.85	4.8175	0.2	Y	TP
7	3.5025	7.698125	3.8	Y	TP
8	2.63	1.2375	−3	N	FP
9	4.085	6.20625	2.19	Y	TP
10	8.3675	8.674375	2.6	Y	TP
11	5.45	4.6225	2.4	Y	TP
12	2.99	2.485	1.8	Y	TP
13	−2.63	4.84	−2.2	N	FP
14	4.925	9.93375	5.4	Y	TP
15	4.8475	5.339375	−4.6	N	FP
16	1.995	4.43375	−0.19	N	FP
17	3.375	6.12625	−3.8	N	FP
18	−1.825	0.72375	0.8	N	FN
19	2.78	0.6125	1.8	Y	TP
20	2.9325	3.050625	0.2	Y	TP
21	3.9025	4.083125	3.8	Y	TP
22	−0.82	−1.56	−1	Y	TN
23	2.6375	2.046875	5.4	Y	TP
24	−1.0325	−2.725625	12	N	FN
25	3.7125	3.270625	3.8	Y	TP
26	2.1725	−0.814375	5.1	Y	TP
27	1.62	0.6625	−0.6	Y	TN
28	8.1825	12.930625	2.6	Y	TP
29	4.7325	3.115625	5.4	Y	TP
30	2.175	0.36125	−0.3	N	FP
31	6.82	5.5125	4.6	Y	TP
32	5.965	7.65875	−1	N	FP
33	7.4	4.15	5	Y	TP
34	3.3875	3.984375	1.2	Y	TP
35	−2.115	−3.85375	4.6	N	FN
36	−1.275	−3.07375	5	N	FN
37	0.165	−7.59125	12.6	N	FN
38	1.5275	4.664375	−2	N	FP
39	0.7275	0.421875	3	N	FN
40	5.2	6	1.18	Y	TP
41	−2.855	−2.35125	2.6	N	FN
42	0.1625	−2.113125	6.6	N	FN
43	1.3025	−1.206875	1.11	N	FN
44	2.6625	3.528125	1.01	Y	TP
45	4.9025	8.370625	−0.2	N	FP
46	3.2375	4.546875	3.37	Y	TP
47	3.79	3.8925	−1.8	N	FP
48	0.2925	3.190625	−3	N	FP
49	3.2375	8.636875	−0.19	N	FP
50	−0.1725	−3.213125	5.8	N	FN
51	4.875	6.42375	1.04	Y	TP
52	0.2375	−1.103125	1	N	FN
53	−2.97	−4.3175	1.4	N	FN
54	4.025	0.58375	−2.2	N	FP
55	2.7	3.565	5.8	Y	TP
56	0.64	5.3125	−1.8	N	FP
57	2.495	2.80875	7.4	Y	TP
58	2.155	−0.08125	4.6	Y	TP
59	1.46	2.105	1	Y	TP
60	1.2875	1.199375	8.2	N	FN

Legend: * A matching result occurred in three cases: when UPCBCT analysis indicates MTD and PI indicates MTD in the premolar region (IP); when UPCBCT analysis indicates MTD and PI indicates MTD in the molar region (IM); when UPCBCT analysis indicates MTD and PI indicates MTD both in the premolar (IP) and molar (IM) regions. ** TP: true positive; FP: false positive; TN: true negative; FN: false negative.

**Table 2:** Test results of Pont’s analysis compared to the reference standard Penn CBCT.

Pont’s Analysis Compared to Penn CBCT	Gold Standard		
Diagnostic test	CBCT Positive	CBCT Negative	Total
Pont Positive	True positive 29	False positive 15	44
Pont Negative	False negative 13	True negative 3	16
Total	42	18	60

**Table 3:** Summary of the accuracy indicators of Pont’s analysis based on the contingency table (Table 2).

Accuracy Indicator	Value	Upper and Lower Limits (Confidence Interval: 95%)
Sensitivity	69%	52.91–82.38%
Specificity	16.6%	3.58–41.42%
PPV	65%	59.14–72.08%
NPV	18.75%	6.95–41.61%
LR+	0.83	0.62–1.11%
LR−	1.86	0.6–5.73%
Accuracy	53.28%	40–66.33%

Legend: PPV: positive predictive value; NPV: negative predictive value; LR+: positive likelihood ratio; LR−: negative likelihood ratio.

**Table 4:** McNemar’s test.

**Case Number**	**Pont’s Index**	**Penn CBCT**	**Pretest-Posttest**
1	0	0	0
2	1	1	0
3	1	0	1
4	1	1	0
5	1	1	0
6	1	1	0
7	1	1	0
8	1	0	1
9	1	1	0
10	1	1	0
11	1	1	0
12	1	1	0
13	1	0	1
14	1	1	0
15	1	0	1
16	1	0	1
17	1	0	1
18	0	1	−1
19	1	1	0
20	1	1	0
21	1	1	0
22	0	0	0
23	1	1	0
24	0	1	−1
25	1	1	0
26	1	1	0
27	0	0	0
28	1	1	0
29	1	1	0
30	1	0	1
31	1	1	0
32	1	0	1
33	1	1	0
34	1	1	0
35	0	1	−1
36	0	1	−1
37	0	1	−1
38	1	0	1
39	0	1	−1
40	1	1	0
41	0	1	−1
42	0	1	−1
43	0	1	−1
44	1	1	0
45	1	0	1
46	1	1	0
47	1	0	1
48	1	0	1
49	1	0	1
50	0	1	−1
51	1	1	0
52	0	1	−1
53	0	1	−1
54	1	0	1
55	1	1	0
56	1	0	1
57	1	1	0
58	1	1	0
59	1	1	0
60	0	1	−1

Legend: 0 = negative test result; 1 = positive test result; false positive (1 → 0) = 15; false negative (0 → 1) = 13; Chi-square (test statistic) = 0.035714286; *p*-value = 0.850106739 → *p* > 0.05 not statistically significant.

## Data Availability

The data presented in this study are available on request from the corresponding author.

## Appendix A

**Table A1:** Summary of the CBCT characteristics.

Ca se Number	CBCT I mAge Quality Infor mAtion
1	Ø20.2 × 20.2 cm (504 × 504 × 504) 400 μm 90 KV 12 mA 4.678 s
2	Ø20.3 × 20.3 cm (507 × 507 × 507) 400 μm 90 KV 12 mA 13.746 s
3	Ø20.2 × 20.2 cm (504 × 504 × 504) 400 μm 88 KV 11 mA 4.716 s
4	Ø20.1 × 20.1 cm (503 × 503 × 503) 400 μm 85 KV 8 mA 4.73 s
5	Ø20.0 × 20.0 cm (501 × 501 × 501) 400 μm 90 KV 12 mA 13.857 s
6	Ø20.1 × 20.1 cm (503 × 503 × 503) 400 μm 84 KV 10 mA 13.764 s
7	Ø20.1 × 20.1 cm (502 × 502 × 502) 400 μm 88 KV 6 mA 4.701 s
8	Ø20.0 × 20.0 cm (500 × 500 × 500) 400 μm 87 KV 12 mA 4.675 s
9	Ø20.1 × 20.1 cm (502 × 502 × 502) 400 μm 88 KV 10 mA 4.747 s
10	Ø20.1 × 20.1 cm (502 × 502 × 502) 400 μm 90 KV 8 mA 13.865 s
11	Ø20.2 × 20.2 cm (504 × 504 × 504) 400 μm 88 KV 11 mA 4.72 s
12	Ø10.0 × 10.0 cm (250 × 250 × 250) 400 μm 90 KV 7 mA 4.074 s 256.0mGyxcm^2^
13	Ø20.0 × 20.0 cm (500 × 500 × 500) 400 μm 82 KV 8 mA 13.55 s
14	Ø20.2 × 20.2 cm (504 × 504 × 504) 400 μm 88 KV 9 mA 13.8 s
15	Ø20.1 × 20.1 cm (502 × 502 × 502) 400 μm 86 KV 8 mA 4.5 s
16	Ø16.0 × 16.0 cm (800 × 800 × 800) 200 μm 99 KV 8 mA 9.0 s 1610.0mGyxcm^2^
17	Ø20.2 × 20.2 cm (504 × 504 × 504) 400 μm 86 KV 11 mA 4.691 s
18	Ø20.1 × 20.1 cm (502 × 502 × 502) 400 μm 89 KV 11 mA 13.435 s
19	Ø20.0 × 20.0 cm (501 × 501 × 501) 400 μm 80 KV 11 mA 4.699 s
20	Ø20.2 × 20.2 cm (504 × 504 × 504) 400 μm 88 KV 11 mA 4.72 s
21	Ø20.1 × 20.1 cm (503 × 503 × 503) 400 μm 84 KV 9 mA 6.4 s
22	Ø20.1 × 20.1 cm (502 × 502 × 502) 400 μm 90 KV 10 mA 4.875 s
23	Ø20.0 × 20.0 cm (500 × 500 × 500) 400 μm 85 KV 11 mA 4.575 s
24	Ø20.2 × 20.2 cm (504 × 504 × 504) 400 μm 90 KV 10 mA 11.8 s
25	Ø20.1 × 20.1 cm (503 × 503 × 503) 400 μm 85 KV 10 mA 7.4 s
26	Ø20.1 × 20.1 cm (502 × 502 × 502) 400 μm 88 KV 11 mA 13.747 s
27	Ø20.3 × 20.3 cm (507 × 507 × 507) 400 μm 90 KV 12 mA 12.646 s
28	Ø20.1 × 20.1 cm (502 × 502 × 502) 400 μm 88 KV 7 mA 5.701 s
29	Ø20.0 × 20.0 cm (500 × 500 × 500) 400 μm 88 KV 9 mA 13.675 s
30	Ø20.0 × 20.0 cm (501 × 501 × 501) 400 μm 89 KV 11 mA 9.457 s
31	Ø20.1 × 20.1 cm (502 × 502 × 502) 400 μm 87 KV 8 mA 9.701 s
32	Ø20.2 × 20.2 cm (504 × 504 × 504) 400 μm 87 KV 10 mA 9.8 s
33	Ø20.0 × 20.0 cm (501 × 501 × 501) 400 μm 87 KV 9 mA 8.457 s
34	Ø20.1 × 20.1 cm (503 × 503 × 503) 400 μm 88 KV 11 mA 9.4 s
35	Ø20.1 × 20.1 cm (502 × 502 × 502) 400 μm 87 KV 9 mA 4.8476 s
36	Ø20.3 × 20.3 cm (507 × 507 × 507) 400 μm 90 KV 12 mA 11.896 s
37	Ø20.2 × 20.2 cm (504 × 504 × 504) 400 μm 88 KV 10 mA 7.886 s
38	Ø20.1 × 20.1 cm (502 × 502 × 502) 400 μm 89 KV 9 mA 7.788 s
39	Ø20.0 × 20.0 cm (500 × 500 × 500) 400 μm 90 KV 11 mA 4.745 s
40	Ø20.0 × 20.0 cm (501 × 501 × 501) 400 μm 88 KV 10 mA 7.885 s
41	Ø20.1 × 20.1 cm (503 × 503 × 503) 400 μm 86 KV 9 mA 9.55 s
42	Ø20.1 × 20.1 cm (502 × 502 × 502) 400 μm 87 KV 8 mA 5.767 s
43	Ø20.2 × 20.2 cm (504 × 504 × 504) 400 μm 87 KV 9 mA 6.89 s
44	Ø20.1 × 20.1 cm (502 × 502 × 502) 400 μm 88 KV 11 mA 13.47 s
45	Ø20.1 × 20.1 cm (502 × 502 × 502) 400 μm 90 KV 10 mA 8.387 s
46	Ø20.0 × 20.0 cm (501 × 501 × 501) 400 μm 89 KV 12 mA 13.765 s
47	Ø20.0 × 20.0 cm (501 × 501 × 501) 400 μm 90 KV 11 mA 4.885 s
48	Ø20.1 × 20.1 cm (503 × 503 × 503) 400 μm 85 KV 10 mA 7.4 s
49	Ø20.1 × 20.1 cm (502 × 502 × 502) 400 μm 90 KV 9 mA 8.797 s
50	Ø20.1 × 20.1 cm (502 × 502 × 502) 400 μm 90 KV 10 mA 6.747 s
51	Ø20.1 × 20.1 cm (502 × 502 × 502) 400 μm 88 KV 10 mA 4.887 s
52	Ø20.1 × 20.1 cm (503 × 503 × 503) 400 μm 84 KV 10 mA 5.34 s
53	Ø10.0 × 10.0 cm (250 × 250 × 250) 400 μm 89 KV 8 mA 5.066 s
54	Ø20.0 × 20.0 cm (500 × 500 × 500) 400 μm 87 KV 9 mA 4.545 s
55	Ø20.3 × 20.3 cm (507 × 507 × 507) 400 μm 90 KV 11 mA 10.556 s
56	Ø20.1 × 20.1 cm (502 × 502 × 502) 400 μm 87 KV 6 mA 4.134 s
57	Ø20.2 × 20.2 cm (504 × 504 × 504) 400 μm 88 KV 9 mA 12.876 s
58	Ø20.0 × 20.0 cm (501 × 501 × 501) 400 μm 90 KV 12 mA 12.757 s
59	Ø20.1 × 20.1 cm (502 × 502 × 502) 400 μm 88 KV 11 mA 8.767 s
60	Ø20.1 × 20.1 cm (502 × 502 × 502) 400 μm 87 KV 8 mA 6.877 s
